# Effects of sand fixation forest restoration on soil water infiltration capacity in Mu Us Sandy Land

**DOI:** 10.3389/fpls.2025.1722655

**Published:** 2026-01-02

**Authors:** Wang Xin, Qin Fucang, Yang Zhenqi, Guo Jianying, Zhen Li

**Affiliations:** 1 College of Desert Control Science and Engineering, Inner Mongolia Agricultural University, Hohhot, Inner Mongolia, China; 2 Ecological Restoration Department, Ministry of Water Resources Pastoral Area Water Conservancy Science Research Institute, Hohhot, Inner Mongolia, China; 3 Inner Mongolia Academy of Forestry Sciences, Hohhot, Inner Mongolia, China; 4 Inner Mongolia Autonomous Region Forestry and Grassland Seedling Station, Hohhot, Inner Mongolia, China

**Keywords:** Mu Us Sandy Land, water holding, infiltration, soil water content, root biomass

## Abstract

**Introduction:**

The Mu Us Sandy Land has severe soil erosion and a fragile ecological environment. The construction of sand fixation forests has markedly increased vegetation coverage. However, water resource scarcity constrains the sustainable development of the ecosystem. Hence, an urgent challenge is to maintain construction of sand fixation forests while mitigating the high soil-water consumption.

**Methods:**

The research selected sand fixation forests of the same recovery years, natural grassland and unrestored bare land for fixed-interval monitoring of soil moisture, together with measurements of vegetation features, soil physical properties, and water-holding and infiltration experiments for each hydrological layer.

**Results:**

Major findings: (1) *Pinus sylvestris* (PS) sand fixation forests had the greatest integrated water-holding capacity, 1.25 times that of bare land. (2) The effective soil-moisture supply depths were 40 cm, 150 cm, 150 cm and 100 cm for Gressland (GL), *Salix cheilophila* (SC) , PS and Bare land (BL). After the moisture supply, PS showed a large water consumption. From the standpoint of conserving soil moisture, GL was the optimal vegetation type, followed by SC. (3) WHC had a direct, significant negative effect on soil water content (SWC) (p < 0.05); WHC also negatively influenced the initial infiltration rate (IIR); and IIR directly influenced SWC. Results further show that canopy and litter layers of sand fixation forests can replenish soil moisture by altering WHC and IIR, but the high water consumption of trees still keeps SWC at low levels.

**Discussion:**

Therefore, based on comprehensive consideration of sand fixation needs and water conservation, *Salix cheilophila* (SC) performs better in water holding and infiltration promotion, and is more suitable for construction of sand fixation forests in this region. In water-lack areas, shrub-grass mixed forests should be considered for construction of sand fixation forests in the future.

## 1 Introduction

The Mu Us Sandy Land is characterized by severe soil erosion and a fragile ecological environment, making it a critical zone for ecological restoration in China (Zhang et al., 2024a). Since the implementation of the Natural Forest Protection Program and the Grain-for-Green Project, vegetation coverage in the region has increased significantly, yielding notable ecological benefits (Li et al., 2022). *Pinus sylvestris* has been widely promoted for sand fixation and afforestation. Salix cheilophila, a dominant shrub species in the Mu Us Sandy Land, is a typical tree species in sandy areas. It exhibits excellent wind erosion resistance, is drought and salt-alkali tolerant, and has a fast growth rate and a well-developed root system. Meanwhile, it possesses strong sand-fixing and soil-conserving functions. However, the Mu Us Sandy Land receives scarce precipitation with highly uneven seasonal distribution. Water scarcity thus remains the core bottleneck restricting ecological restoration efforts (Zhu et al., 2024). Against this backdrop, the water conservation function of restored vegetation is particularly crucial in maintaining ecosystem health, supporting vegetation growth, and promoting sustainable environmental development (Zhang et al., 2024b). Notably, studies have revealed that afforestation in the region may lead to soil moisture deficits, and in severe cases, the formation of desiccated soil layers (Sui et al., 2011). Such moisture deficits not only directly limit vegetation productivity and threaten ecosystem sustainability but also disrupt regional hydrological cycles, exacerbating ecological degradation (Benjamin et al., 2019). *Pinus sylvestris* and the native dominant shrub *Salix cheilophila* have been widely used in sand fixation afforestation in the Mu Us Sandy Land due to their strong stress resistance, fast growth rate, well-developed root system, and prominent sand-fixing and soil-conserving functions (Huang et al., 2024). As a multilayered ecosystem, the structure of sand fixation forests, such as community composition, biomass, and litter accumulation, varies with successional stages or stand age (An et al., 2025). These changes profoundly influence soil physical properties, such as structure and pore characteristics, leading to marked differences in water holding capacity and soil- and water holding effectiveness among various sand fixation forests (Zhao and Wang, 2024). The canopy, serving as the primary interface between the forest and the atmosphere, modulates ecosystem water cycling by shading to reduce evaporation, intercepting precipitation, and regulating transpiration (Su et al., 2022). In arid and semi-arid regions, rainfall is typically short-duration and low-volume. Canopy interception is the first step in rainfall redistribution, and its water-holding capacity cannot be overlooked when assessing the community’s integrated water-holding capacity (Sandra Grunicke and Bernhofer, 2020). The litter layer is a critical component of forest hydrological processes (Han et al., 2024). It effectively retains rainfall, dissipates raindrop kinetic energy, retards surface runoff, suppresses soil evaporation, promotes water infiltration, and ameliorates soil physicochemical properties (Han et al., 2024). With a high water holding capacity, up to 2–4 times its own dry weight (Hou et al., 2018), the litter layer constitutes the second largest contributor to forest water conservation after the canopy, playing a pivotal role in regulating hydrological processes (Luara et al., 2021). Soil water infiltration is the core process that reveals the regulation and transmission functions of forest soil systems (Liu et al., 2020). Vegetation restoration markedly alters infiltration by modifying surface cover (increasing roughness and erosion resistance) and through root activity (improving soil structure and enhancing porosity) (Guo et al., 2019). In arid and semi-arid regions, forest vegetation generally exhibits higher soil infiltration rates than other land-use types (Wilk et al., 2017). When increased vegetation cover enhances soil permeability, it can raise soil water content and nutrient holding, thereby fostering vegetation growth (Yu et al., 2017). To date, studies on the water holding performance of forest stands in the Mu Us Sandy Land have mostly focused on single layers, such as canopy interception, litter holding, or soil water holding capacity, and lack systematic evaluation and comparative analysis of integrated water holding capacity that incorporates the canopy, litter, and soil layers. Particularly in assessments of overall holding, the contribution of the canopy is often neglected. Against the backdrop of climate change intensifying water-resource pressure in arid and semi-arid regions, a comprehensive understanding of the response mechanisms of water holding capacity across typical community layers, namely canopy, litter, and soil, is essential.

Accordingly, this study takes the Hetongmiao River small watershed in the Mu Us Sandy Land as its research area and selects typical sand fixation plantations together with unrestored native grassland and bare-land plots as comparative objects. Drawing on field investigations and laboratory analyses conducted during the 2022–2024 growing seasons, the research systematically measured and analyzed the key indicators governing the integrated water holding capacity, soil infiltration capacity, and soil water content of each community type. The study aims to: (1) elucidate how sand fixation afforestation alters soil physical properties, overall water holding capacity, and soil infiltration capacity; (2) explore the role of different sand fixation forests in the distribution of precipitation through canopy interception, water holding by the litter layer, and soil water holding, and (3) evaluate the water holding performance and moisture-sustainability of different sand fixation forests, thereby providing a scientific basis for selecting the most water-efficient afforestation model in this region.

### 1.1 Overview of study area

**Figure 1:**
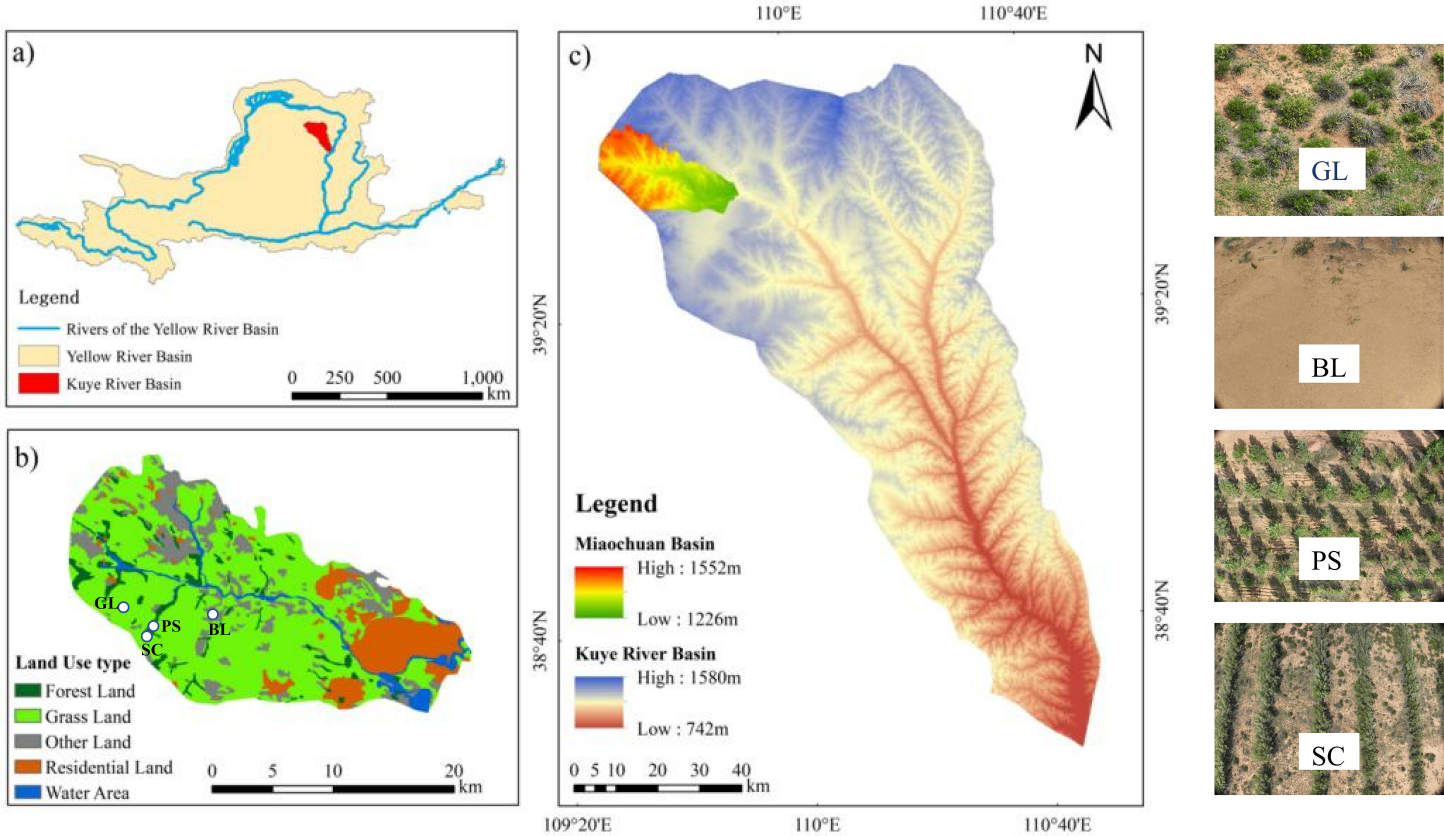
Study area overview and point layout map.

This study was conducted in the Hetongmiao River small watershed, located in the Inner Mongolia section of the Kuye River watershed, a fourth-order tributary of the Yellow River (**Figure 1**). Landsat imagery covering the Hetongmiao River in 2020 was acquired. In ArcGIS, topographic maps were digitized to generate TIN and DEM datasets, from which elevation, slope, and aspect were sequentially extracted. Using ENVI, the remote-sensing images were geometrically corrected, clipped, and then interpreted to derive land-use and fractional vegetation-cover data. The Hetongmiao River small watershed covers 406.15 km². Among these, grasslands occupy the largest share, about 61.92% of the total area. Forest land accounts for 5.05%, and other land for 28.04%. The Hetongmiao River small watershed is characterized by chestnut soil, aeolian sandy soil, sandstone soil, and sand-covered hilly-gully landforms. Meteorological data were obtained from the Hetongmiao River Soil and Water Conservation Monitoring Station (109° 31’ 30.97’’ E, 39° 39’ 2.89’’ N). It lies within an arid-to-semi-arid temperate continental climate zone. The long-term mean annual precipitation is 358.2 mm, with a maximum recorded annual rainfall of 642.7 mm and a minimum of 100.8 mm. Annual sunshine duration averages 2900 h. The effective accumulated temperature ≥10°C is 2751.3°C, while the mean annual evaporation reaches 2563 mm. Prevailing winds are from the northwest, frequently reaching 5–8 on the Beaufort scale; the annual mean wind speed is 3.6 m·s^-1^, with a maximum instantaneous gust of 24 m·s^-1^.

## 2 Materials and methods

### 2.1 Selection and investigation of sample areas

**Figure 2:**
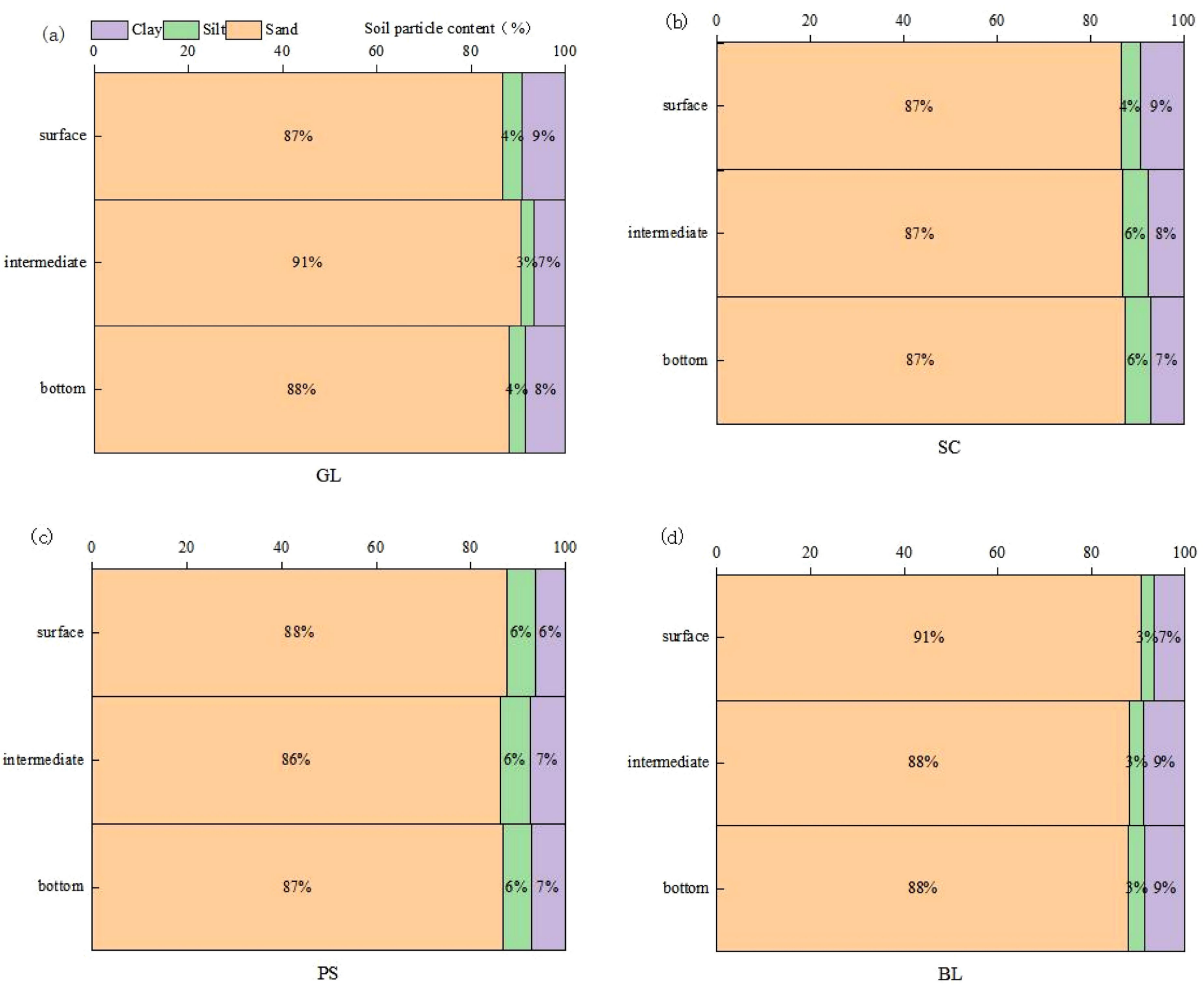
Soil particle constitution in different soil depths. e.g.: **(a)** Soil Particle Constitution in GL, **(b)** Soil Particle Constitution in SC, **(c)** Soil Particle Constitution in PS, **(d)** Soil Particle Constitution in BL.

In 2022, three 15-year-old restored sand fixation plots were selected in the Hetongmiao River small watershed for each of two vegetation types: *Pinus sylvestris* forest and *Salix cheilophila* forest. Three representative plots of the most widespread native grassland were also chosen, together with unvegetated bare-land plots serving as controls. Within each plot, standard individuals were identified through tree/shrub inventory. For *Pinus sylvestris* and *Salix cheilophila* plots, Watch Dog 2800 soil-moisture sensors were installed at depths of 0-10cm, 10-20cm, 20-40cm, 40-60cm, 60-80cm, 80-100cm, 100-150cm, and 150-200cm to continuously monitor the dynamic changes of volumetric soil water content within the 0-200cm soil profile under the canopy. For native grassland and unvegetated bare-sand plots, the sensors were deployed at depths of 0-10cm, 10-20cm, 20-40cm, 40-60cm, and 60-100cm. Data were collected using data loggers, which recorded measurements every 5 minutes. The observation period was the 2024 growing season (May to October), and the data used for plotting soil water content in each soil layer were the average of measurements recorded every 30 minutes. Soil particle-size composition in the surface (0–40 cm), intermediate (40–60 cm), and bottom (60–100 cm) layers was determined in August of each year. We used the USDA Soil Texture Triangle in this study. The contents of clay (< 0.002 mm), silt (0.002–0.05 mm), and sand (0.05-2.0 mm) are shown in **Figure 2**.

### 2.2 Investigation of LAI and biomass

Leaf area index (LAI) was measured on sunny days monthly from May to October of each year using an LAI-2200C plant canopy analyzer (LI-COR, USA). In every plot, 20 permanently marked sampling points were evenly distributed; the mean of these readings was taken as the plot-level LAI. A statistical relationship between LAI and measurement date was then established.

Tree and shrub above-ground biomass: Tree/shrub inventory was conducted within each plot. Based on the inventory data, standard plants were selected, with three representative individuals chosen for each vegetation type. These plants were cut at the base, transported to the laboratory, and oven-dried at 80°C to constant weight to obtain the shrub-layer above-ground biomass for each plot. In October of each measurement year, three 1 m×1 m sub-quadrats were established within every plot. All herbaceous above-ground parts and the entire litter layer within each sub-quadrat were collected, separately oven-dried at 80°C to constant weight and weighed. The mean dry weight was taken as the herb-layer above-ground biomass and the litter-layer biomass for the plot.

Root biomass was determined by direct excavation. In August 2023 and 2024, three representative individuals were selected in each typical plot. Sampling depths were stratified as 0–20 cm, 20–60 cm, and 60–100 cm, consistent with the soil-moisture sampling layers. After sieving, all roots were placed in paper envelopes labeled with plant type and soil depth, then transported to the laboratory. Roots were oven-dried at 65°C for 48 h until constant mass was reached, cooled, and weighed on an analytical balance.

### 2.3 Soil saturated hydraulic conductivity and soil bulk density

In July 2023 and 2024, soil pits were excavated in every plot. Soil cores (100 cm³) were collected with stainless-steel rings at 0–20 cm, 20–60 cm, and 60–100 cm depths to determine bulk density, porosity, and water holding capacity. Saturated hydraulic conductivity (Ks) reflects a soil’s ability to transmit water under saturated, near-saturated, or unsaturated conditions. For Ks measurements, 250 cm³ rings were inserted at the same three depths in each plot, with three replicates per depth. Samples were transported to the laboratory, and Ks was determined by the constant-head method using a KSAT laboratory saturated hydraulic conductivity analyzer.

### 2.4 Litter and soil water holding capacity

Calculation formula of soil water holding capacity:


$${\text{WHC}}_{\text{m}}=10000{\text{hTP}}_{\text{i}}\;$$

WHC=10000hNCP

where: WHC_m_ is the maximum soil water holding capacity (t·hm^-^²); WHC is the soil non-capillary water holding capacity (t·hm^-^²); h is the thickness of the soil layer (m); TP is the total porosity (%); NCP is the soil non-capillary porosity (%).

The water holding capacity of the litter was determined by the immersion method. The weight gain of the litter samples was recorded at successive time intervals (5 min, 10 min, 20 min, 30 min, 40 min, 1 h, 1.5 h, 2 h, 3 h, 4 h, 6 h, 8 h, 12 h, and 24 h) to calculate the water-absorption rate. Litter accumulation was measured using the oven-drying method. Effective interception capacity was then computed with the following formula:


$$\text{WHL}=(0.85{\text{R}}_{\text{M}}-{\text{R}}_{0})\text{xLA}$$

WHL denotes the effective interception capacity of litter, R_0_ represents the natural water content (%), R_M_ is the maximum water holding rate (%), and LA indicates the litter accumulation (t·hm^-²^).

### 2.5 Soil infiltration

Soil infiltration capacity was measured using the double-ring infiltrometer method. At each plot, measurement points were selected beneath both forest canopies and herbaceous patches. Surface weeds and litter were carefully removed to expose the soil surface. Custom stainless-steel rings (inner ring: 20 cm high, 7.5 cm diameter; outer ring: 20 cm high, 15.5 cm diameter) were driven vertically and evenly into the soil to a depth of 10 cm. Water was added until a constant 5 cm head was maintained in both rings, and a Mariotte bottle was used to supply water continuously while keeping the heads constant. During the 90 min infiltration period, the volume of water added to the inner ring was recorded at the following intervals: every 30 s for the first 2 min, every 1min from 2 to 12 min, every 2 min from 12 to 22 min, and every 5 min from 22 to 97 min. The initial infiltration rate was taken as the rate recorded during the previous minute, the steady-state infiltration rate as the rate at 92 min, and the average infiltration rate as the total cumulative infiltration divided by the total elapsed time. The total infiltration volume was calculated as the cumulative amount of water that infiltrated over the entire 97 min period.

### 2.6 Statistical analysis

Statistical analyses were performed with SPSS 26 using one-way ANOVA to compare the effects of vegetation restoration on soil physical properties and vegetation characteristics. Regression analyses were carried out in OriginPro 2021 to identify relationships between vegetation/soil variables and water holding capacity, infiltration capacity, and soil water content after restoration. All figures were prepared with OriginPro 2021 and Chiplot. To further clarify the direct and indirect influences of sand fixation plantation restoration on soil water content via soil physical properties and vegetation traits, partial Mantel tests were conducted to assess the effects of sand, silt, clay, leaf area index (LAI), above-ground biomass (AGB), below-ground biomass (BGB), litter accumulation (LA), saturated hydraulic conductivity (SHC), bulk density (BD), soil capillary porosity (CP), soil non-capillary porosity (NCP), and total porosity (TP) on water holding capacity, infiltration capacity, and soil water content, thereby disentangling their inter-correlations. Partial least squares path modeling was then implemented with the “plspm” package in R (v4.0.2).

## 3 Results and analysis

### 3.1 Changes in vegetation and soil characteristics after restoration of sand fixation forest

**Figure 3:**
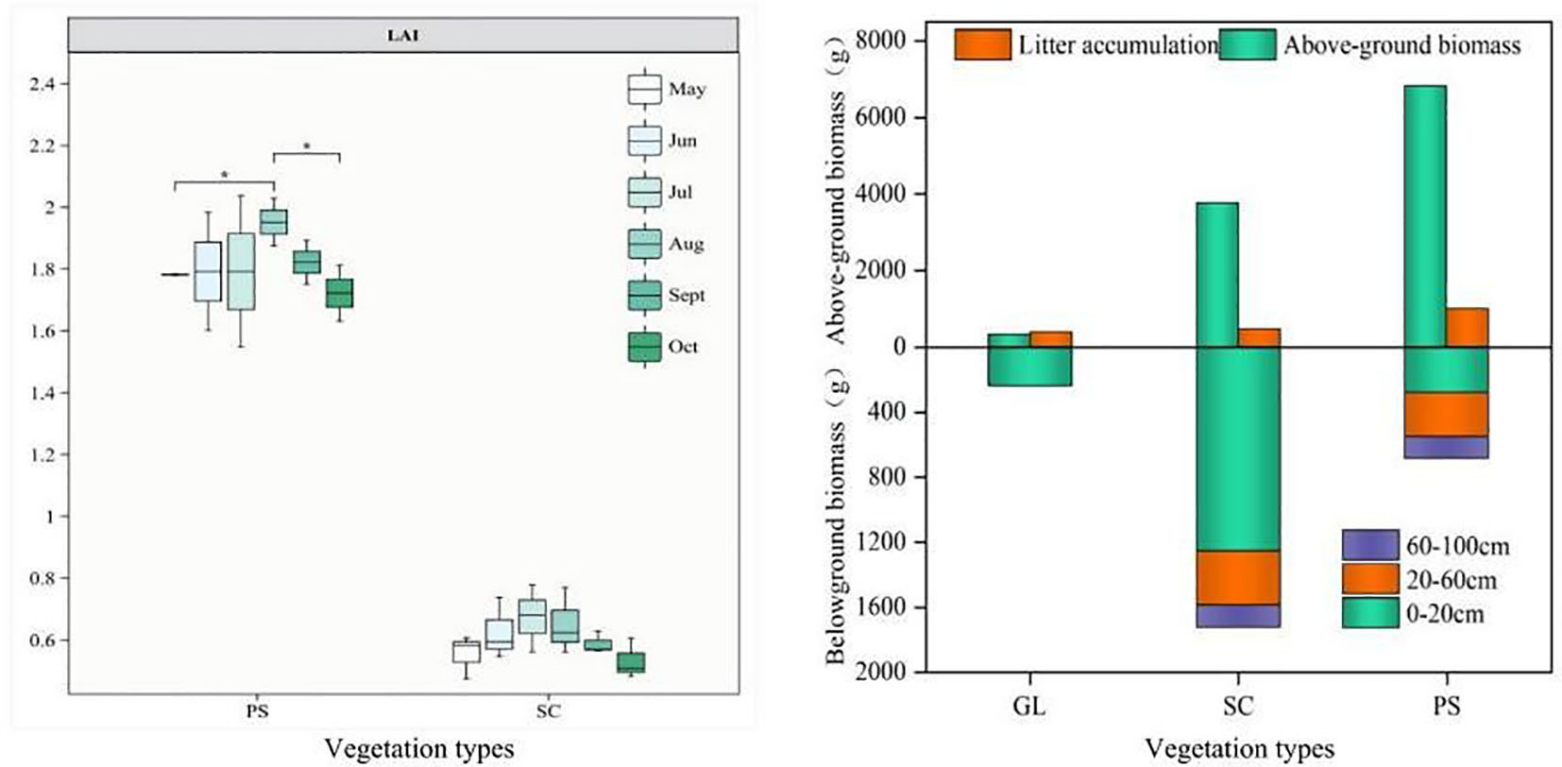
Characteristics of leaf area index and above-ground biomass, below-ground biomass, and litter accumulation in the three vegetation types. GL, grassland; SC, *Salix cheilophila*; PS, *Pinus sylvestris*.

**Figure 4:**
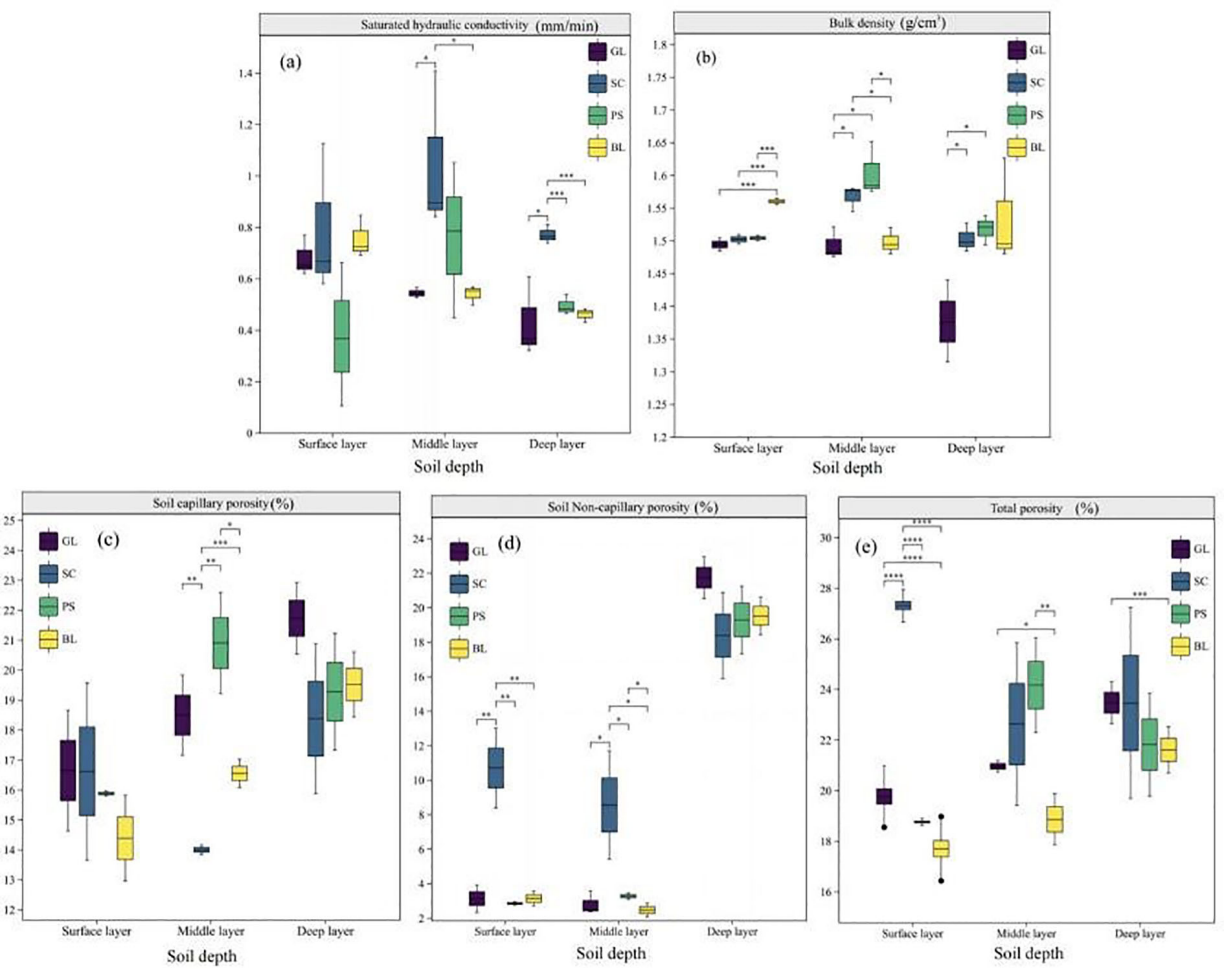
Soil physical characteristics under different vegetation types. e.g.: **(a)** Saturated hydraulic conductivity (SHC), **(b)** Bulk density (BD), **(c)** Soil capillary porosity (CP), **(d)** Soil Non-capillary porosity (NCP), **(e)** Total porosity (TP).

**Figure 3a** shows that the leaf area index (LAI) of PS (1.55-2.04) is significantly higher than that of SC (0.47-0.78). For both forests, LAI increases from May to October gradually at first, peaks in July and August, and then declines in September when the vegetation begins to senesce. (**Figure 3b**) indicates that the litter accumulation in PS stands is 2.3 times that in grassland and 2.19 times that in SC. The above-ground biomass of PS is 34 times that of grassland and 1.94 times that of SC. Total below-ground biomass is highest in SC and lowest in grassland. In the 0–20 cm surface layer, root biomass of SC is 4.27 and 3.51 times that of GL and PS, respectively. In the 20–60 cm middle layer, GL has no roots, and root biomass of SC is 1.49 times that of PS. In the 60–100 cm bottom layer, GL is rootless, and root biomass of SC is 1.57 times that of PS. Saturated hydraulic conductivity in PS and SC increases from the surface to the mid-layer and then decreases (**Figure 4a**), reaching mean maxima of 1.05 mm·min and 0.76 mm·min, respectively, in the mid-layer. Ks in GL and BL is highest in the surface layer (0.68 mm·min and 0.75 mm·min, respectively) and then steadily declines with depth. In the surface layer (0–20 cm, (**Figure 4b**), soil bulk density (BD) of BL is significantly higher (*p* < 0.01) than that of SC, PS, and GL, ranging from 1.48 to 1.57 g·cm^-3^. In the mid-layer (20–60 cm), BD ranges from 1.48 to 1.65 g·cm^-3^, with PS significantly higher than the other communities. In the 60–100 cm layer, BD of PS and SC exceeds that of GL, varying from 1.31 to 1.63g·cm^-3^.Compared with BL, the non-capillary porosity (NCP) of SC is 242.72%, 247.64%, and 155.38% higher in the top, mid-, and bottom layers, respectively (**Figure 4d**). Total porosity (TP) in SC is 54.32%, 19.97%, and 8.55% higher in the corresponding layers (**Figure 4e**). Both NCP and TP gains decline with depth. Grassland and PS also show increases in NCP and TP, but they are less pronounced. Soil capillary porosity (CP) rises slightly in the surface layer across all plots, though the increase is not significant (**Figure 4c**).

### 3.2 Water holding capacity and soil infiltration capacity after restoration of sand fixation forest

**Figure 5:**
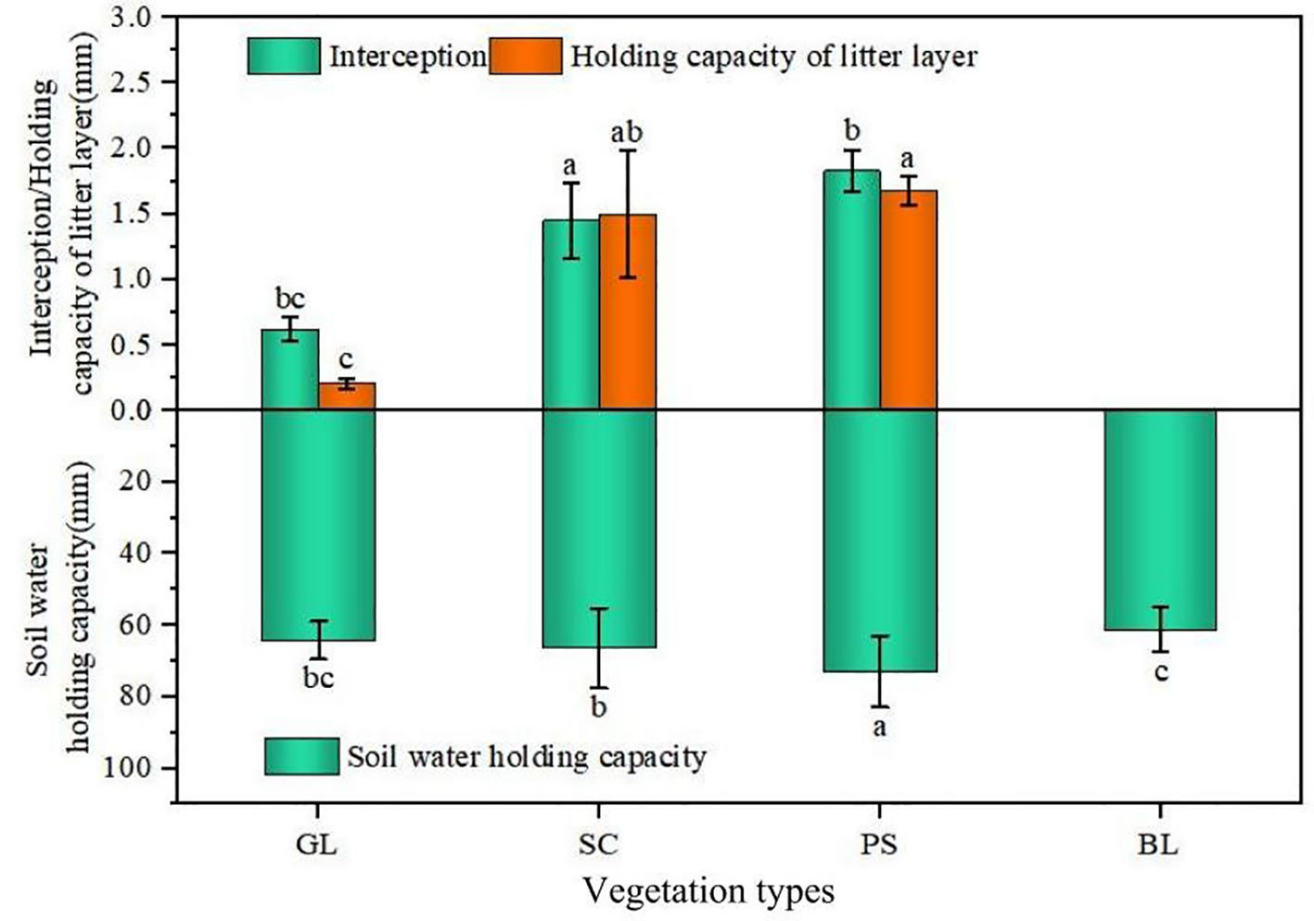
Analysis of comprehensive water holding capacity under different vegetation types. e.g.: Different lowercase letters in the figure indicate significant differences among different sample plots.

**Figure 5** illustrates the canopy interception capacity, litter water holding capacity, and soil water holding capacity across the different vegetation types. Overall, PS exhibited the highest water holding performance in all three components, significantly surpassing the other sites, whereas BL showed the lowest. The integrated canopy water holding capacity is the sum of canopy interception, litter water-holding capacity, and soil water-holding capacity. The integrated canopy water holding capacities of GL, SC, and PS were 1.06, 1.13, and 1.25 times that of BL, respectively.

**Figure 6:**
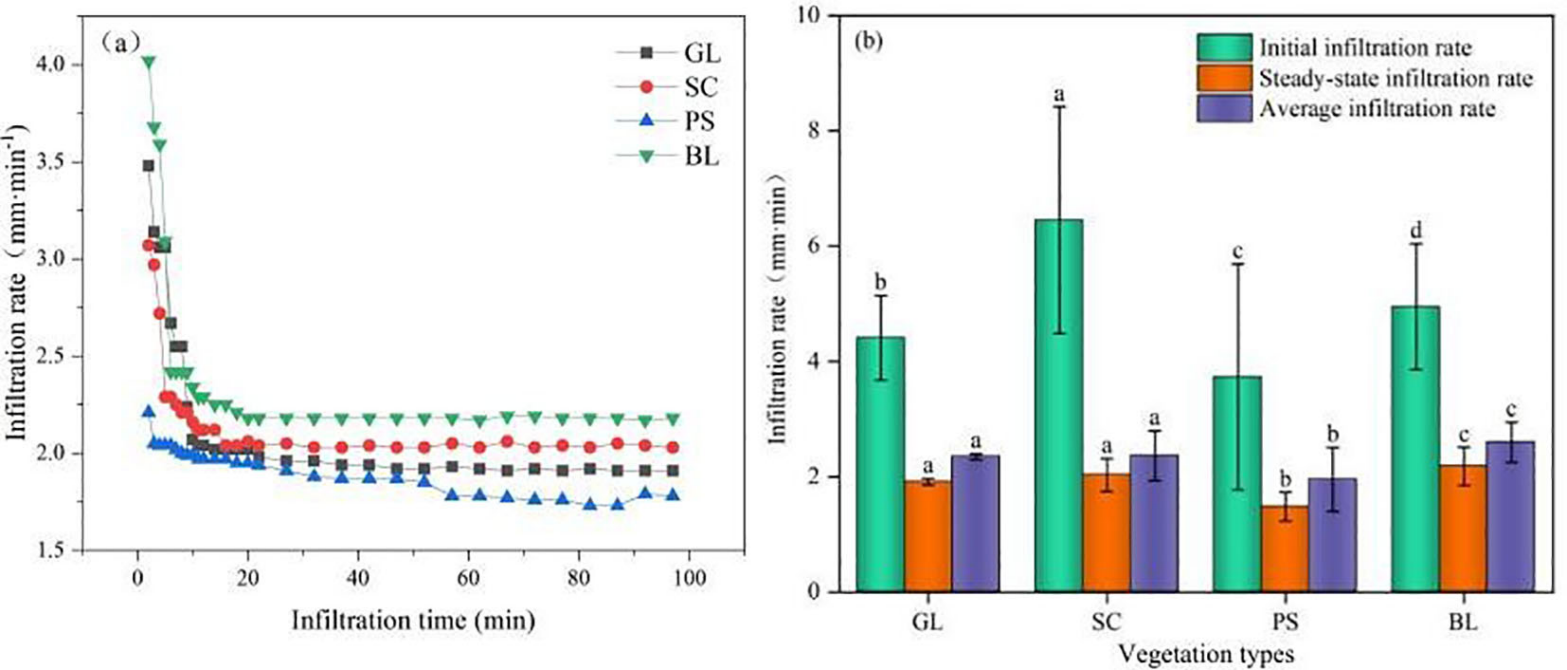
Average infiltration rate under different vegetation types. **(a)** relationships between infiltration time and infiltration rate and **(b)** average infiltration rates. e.g.: Different lowercase letters in the figure indicate significant differences among different sample plots.

The infiltration-rate curves of all vegetation types follow a similar pattern: an initially high rate that declines sharply within the first 2–10 minutes (**Figure 6**). Among the communities, SC exhibits the highest initial infiltration rate, followed by BL and GL, while PS shows the lowest. PS and SC reach steady-state infiltration earliest; after stabilization the ranking is BL > SC > GL > PS. SC’s highest initial infiltration rate is 6.45 mm·min^-1^, which declines rapidly to a stable value of about 2.03 mm·min^-1^, whereas PS’s lowest infiltration rate is 3.73 mm·min^-1^, which declines more gently and stabilizes around 1.78 mm·min^-1^. These results indicate that vegetation cover strongly influences infiltration; different plant communities alter soil structure and surface properties, thereby modifying rainwater entry rates. For all plots, infiltration slows markedly after about 20 min, as the soil approaches saturation, and then levels off at a steady rate.

### 3.3 Diagram of changes in soil water content after restoration of sand fixation forest

**Figure 7:**
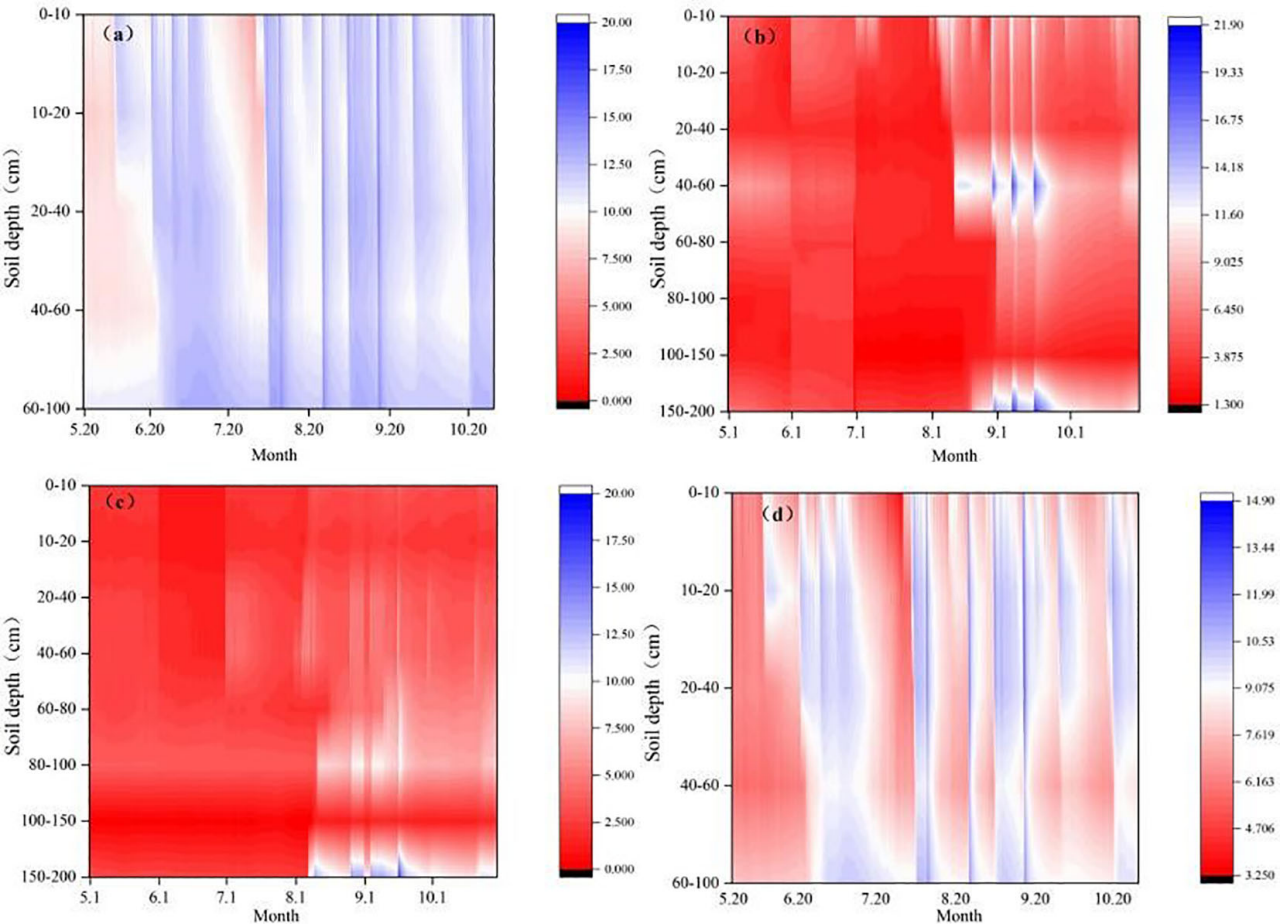
Soil water contents of different vegetation types. e.g.: **(a)** Soil Water Contents of GL, **(b)** Soil Water Contents of SC, **(c)** Soil Water Contents of PS, **(d)** Soil Water Contents of BL.

The initial soil water content of GL in this study was 8.9%, 8.5%, 8.3%, 9.7% and 11.4% in 0-10cm, 10-20cm, 20-40cm, 40-60cm and 60-100cm, respectively. The initial soil water content of SC was 6.1%, 4%, 3.3%, 7.7%, 4.4%, 2.5%, 2.6% and 6% in 0-10cm, 10-20cm, 20-40cm, 40-60cm, 60-80cm, 80-100cm, 100-150cm and 150-200cm, respectively. The initial soil water content of PS in 0-10cm, 10-20cm, 20-40cm, 40-60cm, 60-80cm, 80-100cm, 100-150cm and 150-200cm were 2.3%, 1.7%, 2.7%, 2.7%, 3%, 3.8%, 3% and 3%, respectively. The initial soil water content of BL was 5.9%, 6.5%, 5.8%, 6.7% and 8.4% in 0-10cm, 10-20cm, 20-40cm, 40-60cm and 60-100cm, respectively. In 2024, the soil water content of all vegetation types reached its annual minimum during May–June and then progressively increased, exhibiting pronounced fluctuations that peaked between July and September (**Figure 7**). Variability in soil water content (SWC) was greatest in the surface layer, whereas deeper layers displayed a delayed response to rainfall events. For BL, water recharge penetrated to about 40 cm; subsequent infiltration extended to 60–100 cm, but a discontinuity occurred at 40–60 cm. GL showed deeper recharge than BL, reaching 100 cm. In the SC plots, the recharge depth of SWC initially fluctuated only within the upper 40 cm, but after the rainy season, it increased further, eventually reaching 150 cm. For PS, recharge depths ranged from 100 to 150 cm, and post-recharge SWC in surface layers was markedly lower than in deeper layers.

### 3.4 Correlation analysis of hydrological components and soil water content

**Figure 8:**
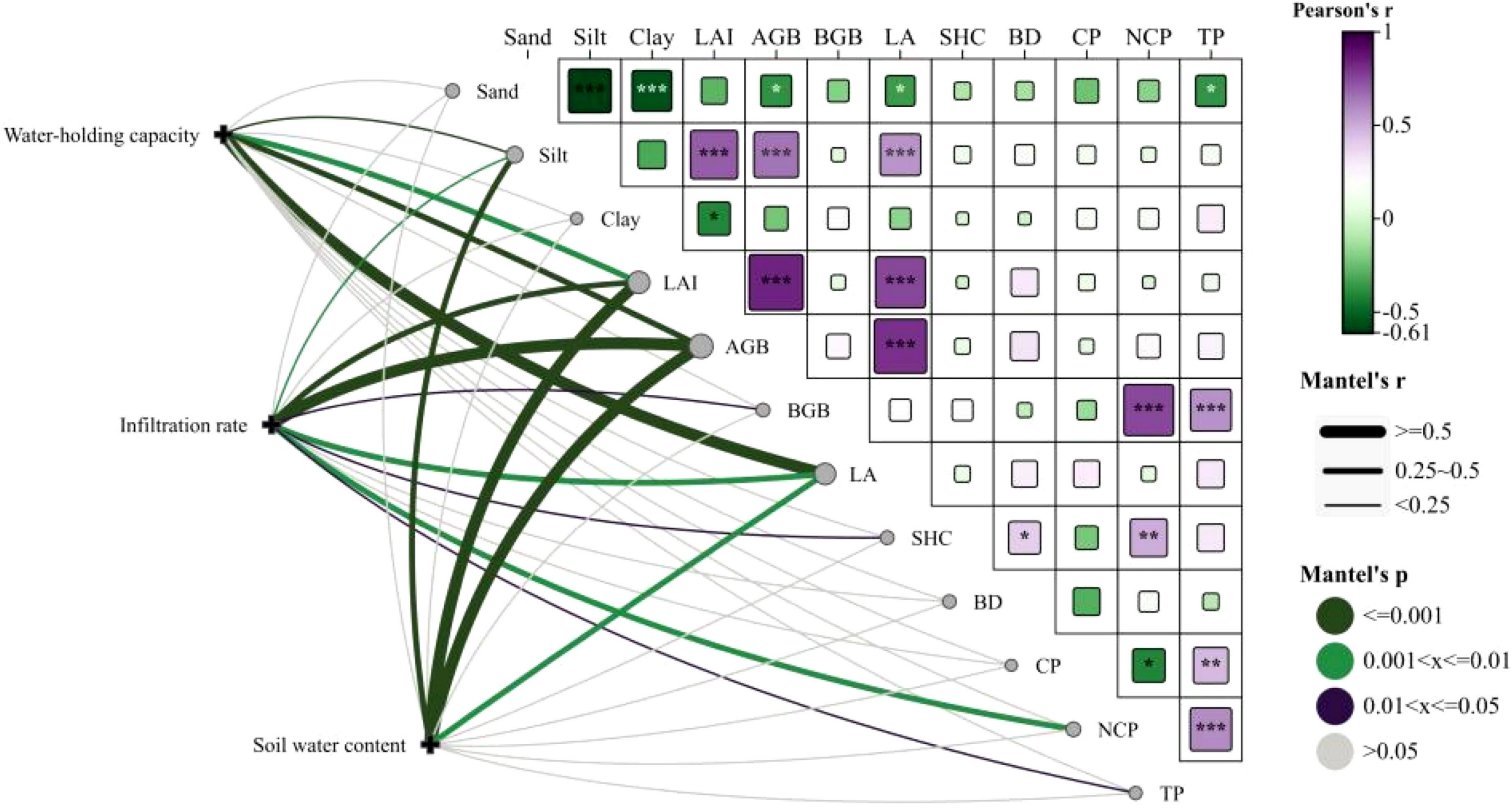
Mantel test of relationships among vegetation traits, soil properties, water holding characteristics, infiltration capacity, and soil water content. e.g.: AGB, above-ground biomass; BGB, below-ground biomass; LA, litter accumulation; SHC, saturated hydraulic conductivity; BD, bulk density; CP, soil capillary porosity; NCP, soil non-capillary porosity; TP, total porosity.

**Figure 8** reveals that water holding capacity is significantly negatively correlated with Silt, LAI, AGB, and LA. Soil infiltration capacity is significantly negatively correlated with Silt, LAI, AGB, LA, and NCP, but significantly positively correlated with BGB, SHC, and TP. Soil water content is significantly negatively correlated with Silt, LAI, AGB, and LA.

**Figure 9:**
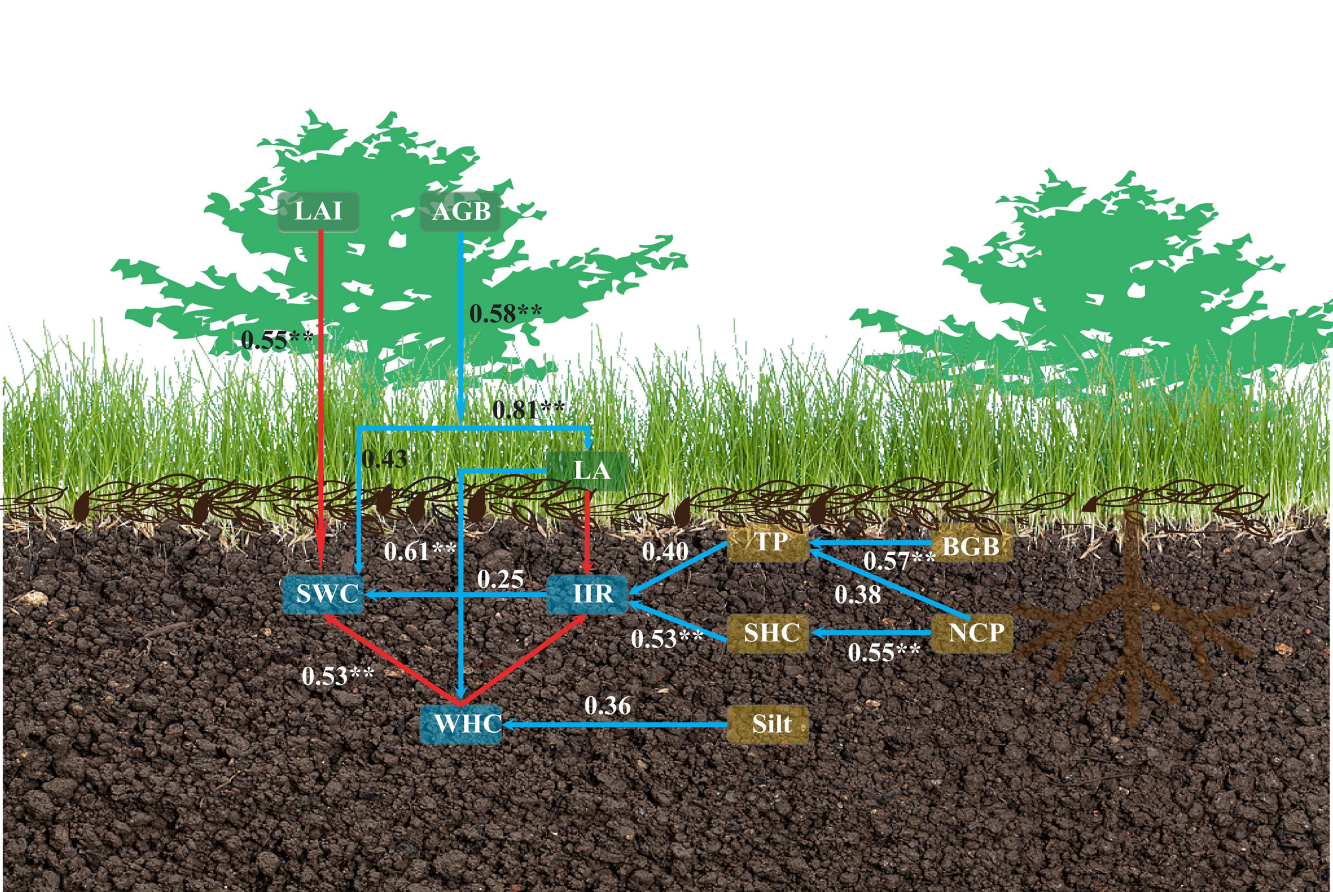
Structural equation model affecting water holding capacity, infiltration capacity, and soil water content.

The structural equation model identified the direct and indirect effects of each factor on water holding capacity (WHC), infiltration rate (IIR), and soil water content (SWC) (**Figure 9**). Above-ground biomass (AGB) exerted a significant positive direct influence on below-ground biomass (BGB) and litter accumulation (LA), thereby indirectly enhancing WHC (*p* < 0.05). Litter accumulation (LA) had a significant positive direct effect on WHC but a significant negative direct effect on IIR (*p* < 0.05). Non-capillary porosity (NCP) directly influenced saturated hydraulic conductivity (SHC). SHC, in turn, significantly and positively affected IIR directly (*p* < 0.05). NCP, leaf area index (LAI), and AGB indirectly influenced IIR. Both LAI and AGB exhibited significant negative direct effects on SWC (*p* < 0.05). Among WHC, IIR, and SWC, WHC exerted a significant negative direct effect on SWC (*p* < 0.05), WHC negatively influenced IIR directly, and IIR, in turn, directly impacted SWC.

## 4 Discussion

### 4.1 Vegetation effects on water input partitioning

Canopy interception represents a net loss in rainfall redistribution, and its magnitude directly governs the amount of effective precipitation reaching the forest floor (David et al., 2006). In this study, PS plantations exhibited the strongest canopy interception capacity, followed by SC. Canopy interception is controlled by the water holding characteristics of foliage (Li et al., 2025). Leaves with larger surface areas generally possess greater water holding potential, require longer times to reach saturation, and thus yield higher canopy interception volumes (Roser et al., 2020). Owing to its low canopy height, small canopy volume, and prostrate branching habit (Ran et al., 2022), SC has a lower interception capacity than PS. Moreover, the rough bark surface and needle-shaped leaves of PS enhance surface tension on the canopy (REYNOLDS, 1965), thereby increasing its canopy storage capacity (Magliano et al., 2019).

The litter layer serves as a critical bridge between above-ground vegetation and the soil, supplying nutrients for plant growth and playing a key role in water conservation (Niu et al., 2024). Litter cover markedly reduces surface runoff and effectively suppresses soil erosion (Liu et al., 2017). It also ameliorates surface microclimates by increasing humidity, lowering temperature, and decreasing evaporative losses (Jourgholami et al., 2022; Cui and Pan, 2023; Bulcock and Jewitt, 2012). However, the litter water holding capacities recorded in this study for GL, SC, and PS plots (0.21-1.68 mm) are significantly lower than those reported for beech, hornbeam, and maple forests in northern Iran (84.1, 59.16, and 25.81 mm, respectively) (Meghdad et al., 2021). This disparity is primarily attributed to the much slower decomposition rate of coniferous litter compared to deciduous and broad-leaved evergreen species, a consequence of its lower litter quality (Zhang et al., 2019).

Overall, results show that the PS plantation has the highest total water holding capacity, followed by the SC, while the BL has the lowest. The outstanding integrated water holding performance of PS is mainly attributed to its more canopy, litter, greater above-ground biomass, and soil structure. On the one hand, this promotes greater litter input and its subsequent transformation into soil organic matter; on the other hand, the intense root activity secretes more cementing substances, facilitating soil aggregate formation, improving soil structure, increasing porosity, and enhancing aeration, thereby ultimately leading to a markedly higher water holding capacity. In contrast, the bare land lacks vegetation cover and a litter layer, so its nutrient cycling is impaired and its soil structure remains poor, resulting in the weakest water holding capacity.

### 4.2 Soil structural modification pathways

Soil particles, as the fundamental units of soil structure, markedly influence soil water regimes and bulk-density variations (Chen et al., 2019). Compared with BL, the silt and clay contents in GL, SC and PS plots increased, indicating that vegetation restoration drives soil texture toward finer fractions (Meng et al., 2025). Moreover, along the vertical profile, surface soils were generally finer than deeper layers, a pattern consistent with the findings of Li (2018). In the study, surface-soil (0–20 cm) bulk density in GL, SC and PS was significantly lower than in BL, probably because plant roots are concentrated mainly within the 0–40 cm horizon. Notably, the depth-wise trends in bulk density differed among the planted stands: bulk density in grassland and bare land declined with depth, whereas in SC and PS it first increased and then decreased, trends closely linked to root biomass distribution.

Saturated hydraulic conductivity (SHC) is a key indicator of infiltration capacity and the main factor governing hillslope soil-water movement (Abhishe et al., 2023). SHC is closely linked to soil physical properties and land-use types (Guillaume et al., 2023). The results show that above-ground canopy and litter cover effectively reduce raindrop splash erosion, helping the surface soil maintain high SHC values. Compared with GL and BL, the SC plots, characterized by greater canopy cover, a thicker litter layer, and a well-developed root system, exhibited significantly higher SHC, particularly in deeper soil layers (*p* < 0.05). Root activity (growth and turnover) in SC directly creates soil macropores (Hu et al., 2022) and, through organic-matter inputs, enhances aggregate stability and improves soil structure, thereby markedly increasing SHC (Zhang et al., 2024c). Notably, SHC in the 20–60 cm layer was higher than in the 0–20 cm layer for both SC and PS, indicating that mid-layer roots strongly promote SHC, most likely because this horizon experiences less disturbance.

Soil infiltration is the process by which water moves from the surface into the soil, filling pores and migrating until a dynamic equilibrium is reached (Wu et al., 2016). The intensity of infiltration directly affects surface runoff generation and soil erosion (Zhuang et al., 2020). Stronger infiltration capacity means greater soil capacity to absorb and store water (Franzluebbers, 2002), thereby effectively reducing surface runoff and erosion risk (Zhuang et al., 2020). In this study, infiltration processes of 0–97 min were monitored in sand fixation plantations, natural grassland, and bare sand within the Hetongmiao River small watershed. Results showed that PS had the lowest initial, steady-state, and average infiltration rates. This was mainly attributed to a 3% increase in silt content in PS surface soil compared to BL and its lower surface soil porosity, leading to a SHC lower than BL, thereby weakening infiltration capacity. In contrast, the steady-state infiltration rate of SC soil was significantly higher than other sites. This benefit was derived from SC’s substantial root biomass and its well-developed three-dimensional “canopy-surface cover” structure, which effectively buffers raindrop impact, protects surface soil structure from destruction, and creates favorable conditions for water infiltration (Bi et al., 2014). Huang et al. (2024) also emphasized the critical role of roots in promoting preferential flow formation. Steady-state infiltration rate is an important indicator reflecting the ultimate infiltration capacity of soil (Yang et al., 2020). During the overall infiltration process, the initial infiltration rate of all vegetation types was higher than their steady-state rate. This was primarily due to the rapid acceptance and transmission of water by soil pores during the initial infiltration stage (Zhang et al., 2020). As infiltration continued, large soil aggregates tended to disintegrate under water action, producing microaggregate particles that clogged some pore channels, causing the overall infiltration rate to gradually decline until stable water flow paths were formed, at which point the infiltration rate became constant, reaching the steady infiltration stage (Chen et al., 2010). Soil infiltration performance is closely related to its physical properties (Cui et al., 2022). Correlation analysis showed that soil infiltration capacity was significantly positively correlated with below-ground biomass, saturated hydraulic conductivity, and total porosity (*p* < 0.05), and significantly negatively correlated with above-ground biomass, non-capillary porosity, litter accumulation, and sand content (*p* < 0.05). Total porosity is an important parameter measuring soil capacity to hold water and air (Cui et al., 2019). As the main pathway for water migration from the surface to subsurface, higher total porosity generally indicates stronger soil infiltration capacity. Results from the Structural Equation Model constructed in this study further confirmed the significance of this positive relationship.

### 4.3 Soil moisture dynamics across temporal scales

In arid and semi-arid regions where precipitation is scarce and water resources are limited, soil moisture becomes the key ecological factor constraining plant growth and development, profoundly shaping vegetation spatial patterns (Jonathan et al., 2018). Atmospheric precipitation is the primary source of soil-water recharge, and its dynamics are tightly coupled with rainfall events, causing marked differences in water status among dunes with varying degrees of stabilization (Sebastian and Samiro, 2020). Results show that SWC at all depths exhibits consistent monthly fluctuations that closely match both plant phenology and the seasonal rainfall regime in the Mu Us Sandy Land. Specifically, rainfall is low from May to June, whereas rainfall in July to September can account for about 90% of the growing-season total. SWC differs significantly among the GL, SC, PS and BL plots, mainly because of differences in canopy structure and water-use characteristics among vegetation types (Ran et al., 2022; Magliano et al., 2019). During the rainy season and peak-growing period, the above-ground biomass of tree and shrub stands increases, enhancing rainfall interception and simultaneously demanding large amounts of soil water to sustain growth; consequently, their SWC becomes markedly lower than that of GL and BL plots. Moreover, the extensive root systems of planted trees and shrubs intensively deplete soil water, so the 0–150 cm profile is rapidly replenished during the rainy season but is quickly exhausted by vegetation uptake (Li, 2021). During the growing season of *Pinus sylvestris*, especially when water supply is adequate, its vigorous photosynthesis and transpiration lead to the absorption and consumption of substantial amounts of water through its root system. While under drought stress, it can effectively reduce water loss and maintain adequate water in its tissues without excessive consumption (Brenda et al., 2025). Fu et al. (2015) also reported that shrubs exert a greater influence than trees on the vertical distribution of SWC. As found, PS roots are concentrated mainly in the 0–60 cm layer, and SWC within this zone is significantly lower than in the SC plots. This aligns with Tian et al. (2022), who observed that PS roots reduce SWC, and suggests a potential risk of water stress for vegetation restoration in the Mu Us Sandy Land. The result also corroborates Mei (2019), who found that forest water consumption is concentrated in the growing season, leading to lower shallow-soil SWC in tree and shrub stands than in grassland and bare land. In summary, although this study has separately analyzed the water-holding capacity, infiltration characteristics, and dynamic changes of soil moisture of sand fixation forests, it only focused on the changes during a single growing season. Therefore, continuous monitoring is still necessary to reflect the interannual dynamic changes. Based on the above discussions, we found that rainfall is the triggering factor affecting soil moisture, and the impacts of different rainfall types on the replenishment depth of soil moisture still require further analysis through controlled experiments.

## 5 Conclusions

This study evaluated the eco-hydrological effects of different sand fixation plantations in the Mu Us Sandy Land and compared them with unrestored bare land. The main conclusions are as follows:

(1) Field surveys revealed that PS had the largest litter accumulation and above-ground biomass. SC plots possessed the highest below-ground biomass, thus effectively improving soil physical properties. The total porosity (TP) in the surface, mid-, and deep soil layers was significantly increased by 54.32%, 19.97%, and 8.55%, respectively, compared with BL (P < 0.05).
(2) The integrated water holding capacities of GL, SC, and PS were increased by 1.25, 1.13, and 1.06 times, respectively, compared with that of BL. SC exhibited the highest initial infiltration rate (6.45 mm/min), and the steady-state infiltration rates ranked as BL (2.18 mm/min) > SC (2.03 mm/min) > GL (1.91 mm/min) > PS (1.78 mm/min).
(3) Effective soil-water recharge depths for GL, SC, PS, and BL were 40 cm, 150 cm, 150 cm, and 100 cm, respectively. From the perspective of maintaining soil moisture, GL was the best vegetation type.
(4) Structural equation modeling (SEM) revealed that water holding capacity (WHC) was a direct, significant negative factor affecting soil water content (SWC) (P < 0.05); WHC negatively influenced initial infiltration rate (IIR), which in turn directly affected SWC. Vegetation directly or indirectly regulates WHC, IIR, and SWC by influencing water input. Balancing sand-fixation needs with water holding effectiveness, SC performed better in both moisture conservation and infiltration promotion, making it more suitable for the construction of sand fixation plantations in this region.

## Data Availability

The raw data supporting the conclusions of this article will be made available by the authors, without undue reservation.
